# Supplementary figures and images for: Investigating the biosynthesis and roles of the auxin phenylacetic acid during Pseudomonas syringae-Arabidopsis thaliana pathogenesis
Source: Front Plant Sci. 2024 Jul 18;15:1408833. doi: 10.3389/fpls.2024.1408833 (PMC11291249; doi:10.3389/fpls.2024.1408833)

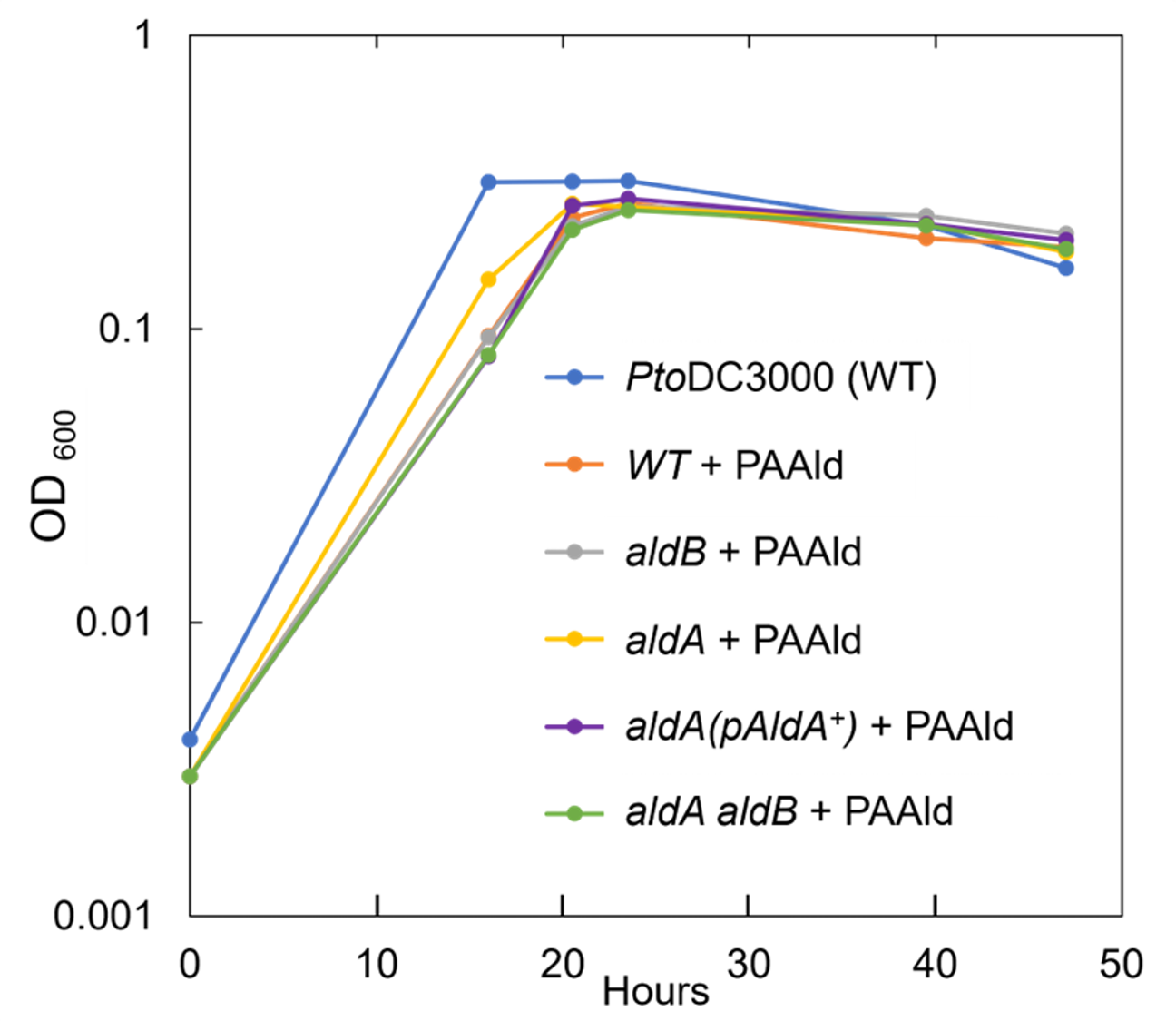

Supplement: Supplementary file 1 [file Image_1.tif]

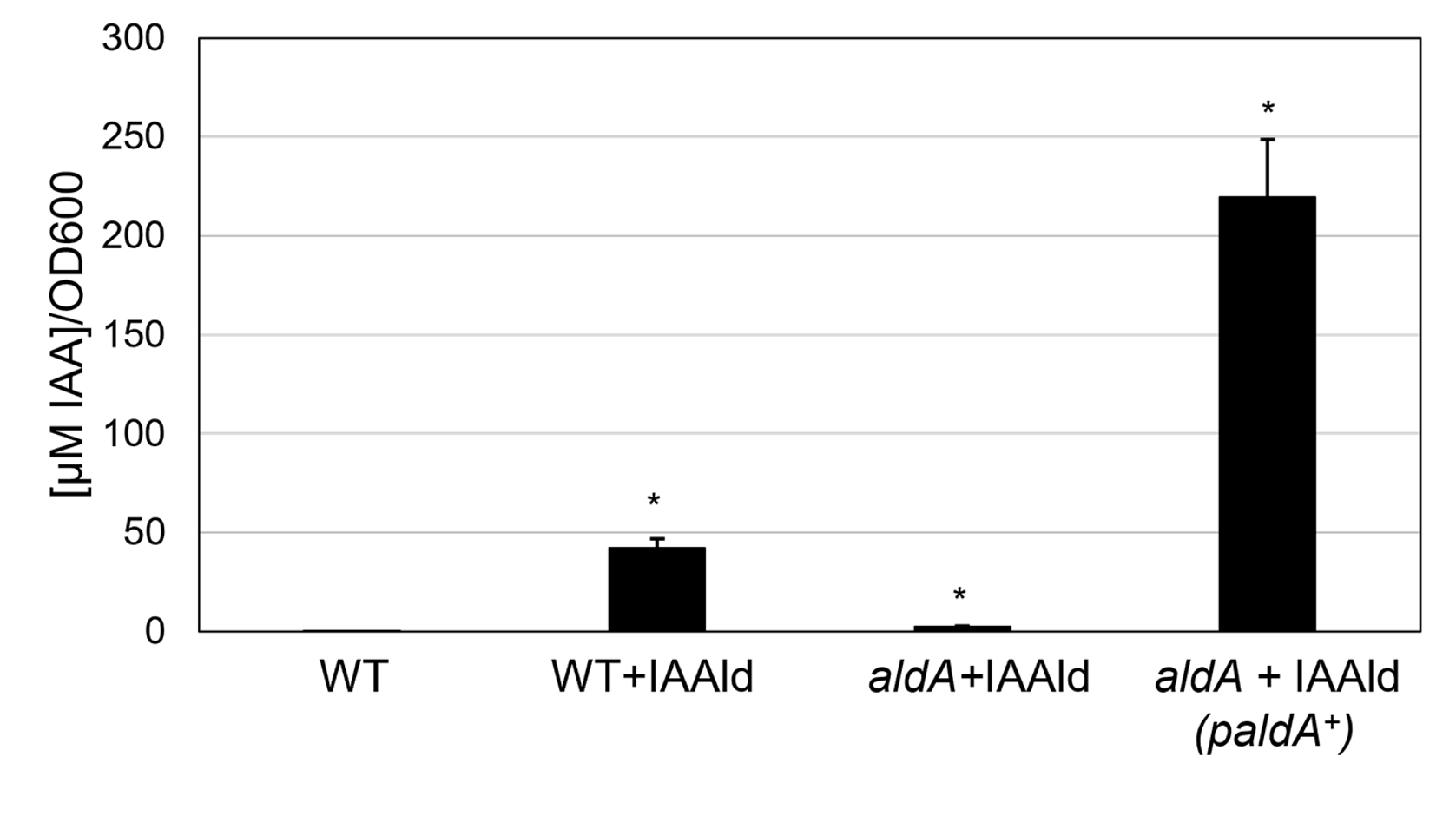

Supplement: Supplementary file 2 [file Image_2.tif]

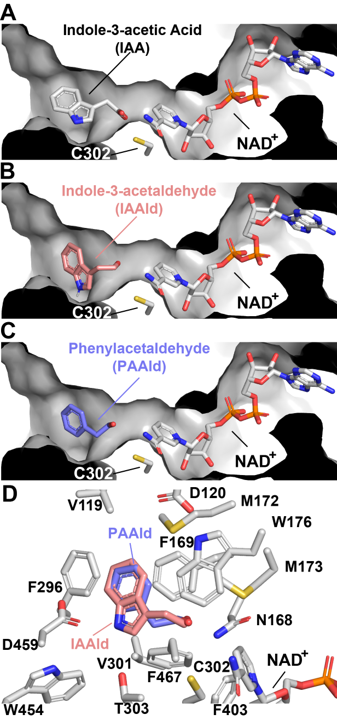

Supplement: Supplementary file 3 [file Image_3.tif]

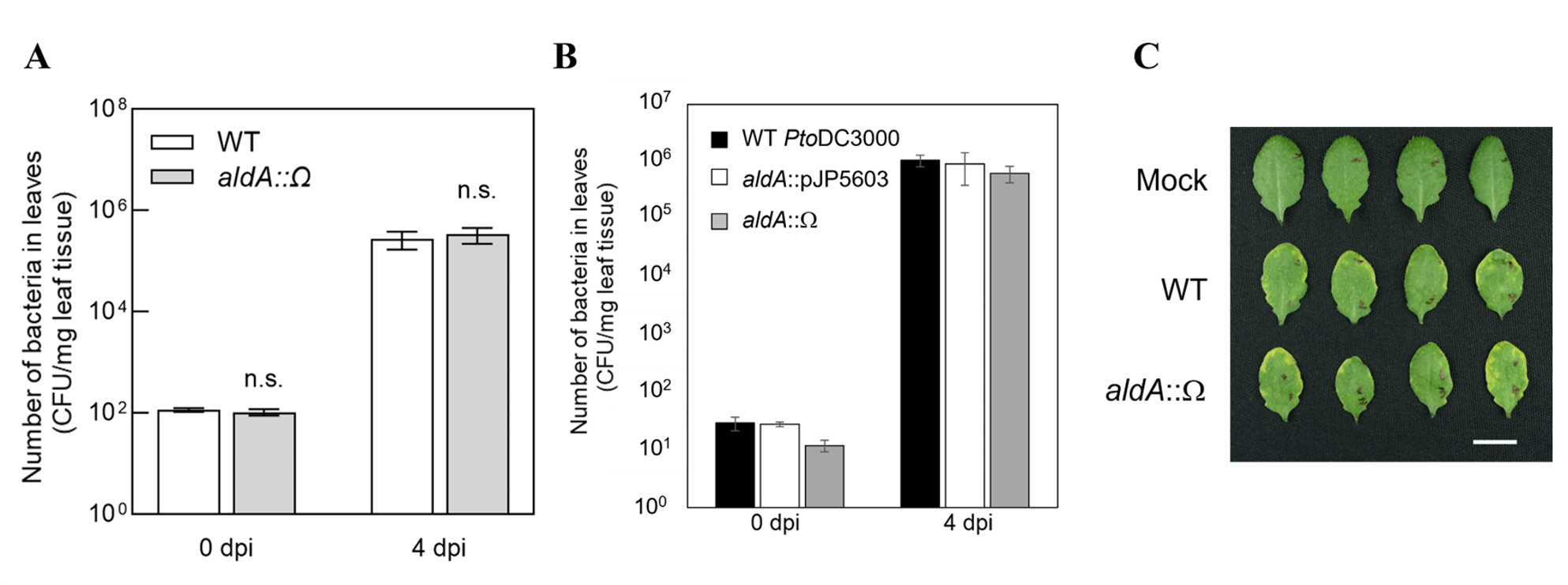

Supplement: Supplementary file 4 [file Image_4.tif]
